# Visualization of Scattered Tumor Regression Following Neoadjuvant Chemoimmunotherapy With a Novel Intraoperative PET/CT Scanner

**DOI:** 10.1002/hed.70333

**Published:** 2026-05-25

**Authors:** Andreja Radevic, Joaquin Austerlitz, Ashtyn McAdoo, Kyrionna Golliday, Takeshi Takahashi, Mitra Mehrad, Adam J. Rosenberg, Barbara A. Murphy, Eben Rosenthal, Michael C. Topf

**Affiliations:** ^1^ Department of Otolaryngology‐Head and Neck Surgery Vanderbilt University Medical Center Nashville Tennessee USA; ^2^ Vanderbilt University Department of Chemical and Physical Biology Nashville Tennessee USA; ^3^ Vanderbilt University Institute of Imaging Science Nashville Tennessee USA; ^4^ Department of Pathology, Microbiology and Immunology Vanderbilt University Medical Center Nashville Tennessee USA; ^5^ Department of Radiology and Radiological Sciences Vanderbilt University Medical Center Nashville Tennessee USA; ^6^ Division of Hematology and Oncology Vanderbilt Ingram Cancer Center Nashville Tennessee USA

**Keywords:** head and neck squamous cell carcinoma, intraoperative PET/CT, neoadjuvant chemoimmunotherapy, pathologic response, tumor regression pattern

## Abstract

**Background:**

Neoadjuvant chemoimmunotherapy is being explored in locally advanced head and neck squamous cell carcinoma (HNSCC), yet the spatial patterns of pathologic response and clinical implications remain poorly characterized.

**Methods:**

A 79‐year‐old female with recurrent left oral tongue SCC (PD‐L122C3 Combined Positive Score 90) who underwent prior resection and free flap reconstruction received neoadjuvant pembrolizumab, paclitaxel, and carboplatin followed by definitive surgical resection with intraoperative positron emission tomography/computed tomography (PET/CT) imaging.

**Results:**

Final histopathology revealed two discrete foci of residual poorly differentiated SCC, measuring 1.8 and 0.5 cm, separated by an 8 mm intervening bridge of inflammation without viable tumor. Intraoperative PET/CT with 3D specimen mapping confirmed the spatial relationship between residual foci and intervening treatment effect.

**Conclusions:**

Pathologic response to neoadjuvant chemoimmunotherapy in HNSCC can manifest as a multifocal regression pattern. Intraoperative real‐time approaches to assessing treatment response warrant further investigation.

**Trial Registration:** ClinicalTrials.gov identifier: NCT06915454

## 1 Introduction

Neoadjuvant immunotherapy and chemoimmunotherapy are increasingly being explored in the treatment of resectable, locally advanced head and neck squamous cell carcinoma (HNSCC) to improve long‐term oncologic outcomes. The phase III KEYNOTE‐689 trial demonstrated improved event‐free survival with perioperative pembrolizumab added to standard of care treatment for resectable, locally advanced HNSCC, supporting a growing role for neoadjuvant immunotherapy in the curative‐intent setting [1, 2, 3].

While these results are promising, there is variability in pathologic response to neoadjuvant immunotherapy in HNSCC. In particular, the spatial patterns of tumor regression following neoadjuvant immunotherapy, and their clinical significance, are only beginning to be characterized [4]. How to classify, interpret, and manage these heterogeneous response patterns remains unanswered. Additionally, preoperative imaging, particularly positron‐emission tomography/computed tomography with [18F] fluorodeoxyglucose (FDG PET/CT), has been shown to be unreliable in predicting pathologic responses to neoadjuvant immunotherapy [5]. Head and neck surgeons will increasingly need to determine the extent of surgical resection in the setting of treatment response yet may not be able to rely on preoperative imaging alone to guide intraoperative surgical decision making. Novel intraoperative imaging modalities, such as micro‐PET/CT, may address this gap by providing real‐time assessment of residual metabolic activity within the surgical specimen.

We present a case of recurrent SCC of the oral tongue in a patient with a combined positive score (CPS) of 90 based on PDL1 immunohistochemistry 22C3 pharmDX who demonstrated a pattern of pathologic response characterized by two foci of residual disease with an intervening inflammatory bridge following neoadjuvant chemoimmunotherapy with pembrolizumab. In this case report, we aim to highlight a unique treatment response and contribute to the understanding of response patterns in the perioperative immunotherapy era. We further discuss the role of intraoperative imaging, limitations of current predictive biomarkers in HNSCC, and the implications of response pattern heterogeneity for treatment planning.

## 2 Case Presentation

A 79‐year‐old female presented to the head and neck surgery clinic 2 years after undergoing left oral cavity composite resection including marginal mandibulectomy, neck dissection, and radial forearm free flap reconstruction for pT4aN1 p16 negative SCC of the left mandibular alveolar ridge with focal bone invasion. Final margins from the index case were negative, and one lymph node was positive for SCC with no extranodal extension (ENE). The patient declined adjuvant radiotherapy. She subsequently presented with a new 2 cm left lateral oral tongue lesion which was biopsied for poorly differentiated invasive keratinizing SCC (PDL1 22C3 CPS 90). A contrast‐enhanced computed tomography (CT) of the neck (Figure 1A) revealed an enhancing 2.2 × 1.7 cm nodule of the left lateral tongue without nodal disease. Left partial glossectomy with repeat free flap reconstruction was discussed, but the patient was hesitant to proceed given her age and difficulty in recovering from the initial operation.

**FIGURE 1:**
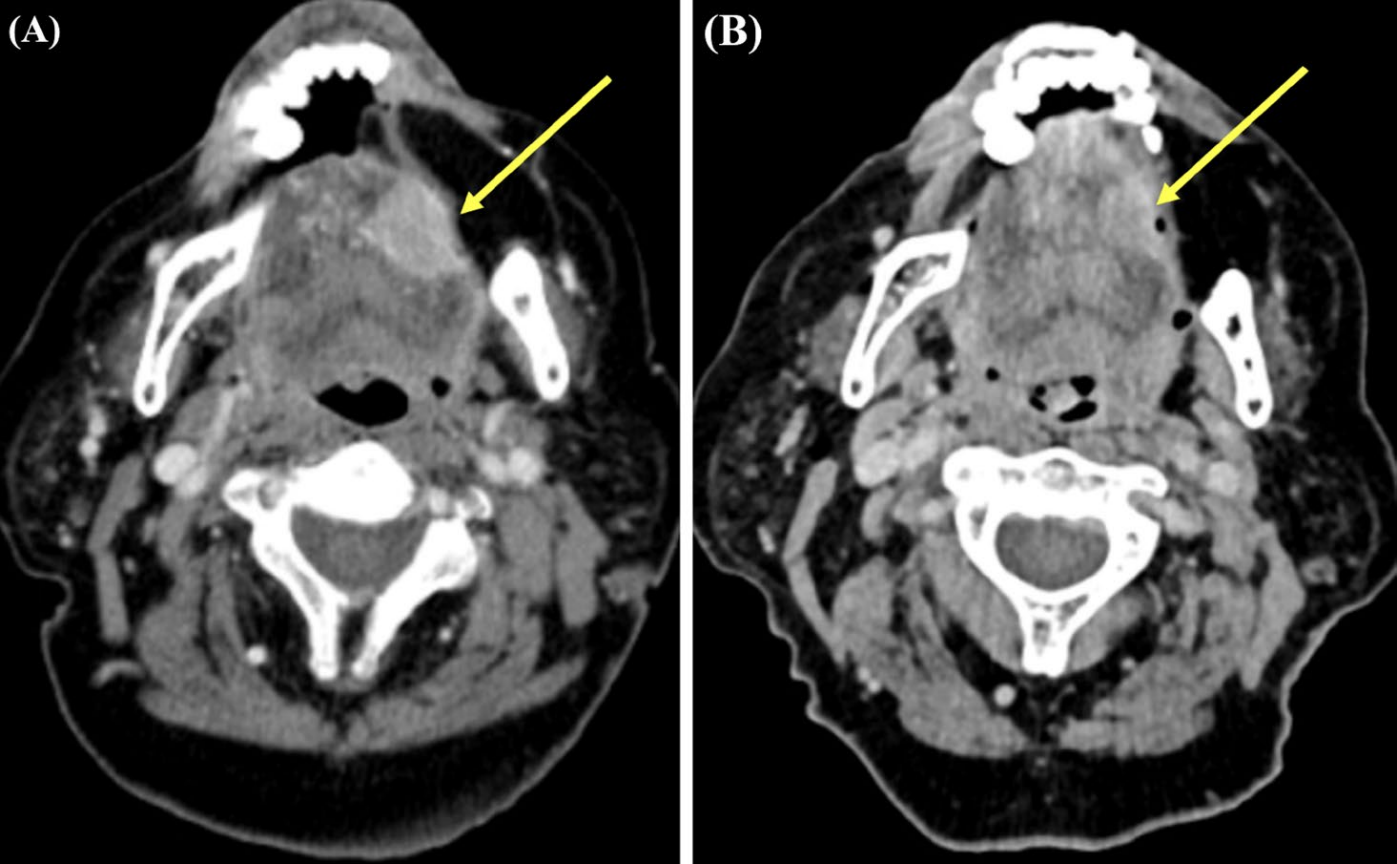
Clinical and radiographic presentation of recurrent left oral tongue squamous cell carcinoma. (A) Pre‐treatment contrast‐enhanced axial CT of the neck revealing a 2.2 × 1.7 cm nodule of the left lateral tongue (yellow arrow). (B) Post‐treatment contrast‐enhanced axial CT of the neck demonstrating interval decrease in tumor size (yellow arrow) following neoadjuvant chemoimmunotherapy.

Given her recurrent disease and hesitancy to proceed directly with resection and free flap reconstruction, the patient underwent induction chemoimmunotherapy with planned interval assessment of response. The patient underwent six cycles of weekly 60 mg/m^2^ paclitaxel and carboplatin (AUC 2) chemotherapy with 200 mg pembrolizumab immunotherapy administered every 3 weeks for two cycles. A post‐treatment contrast‐enhanced neck CT (Figure 1B) demonstrated an interval decrease in tumor size to 1.8 × 1.1 cm. Following reassessment, the patient proceeded with surgical resection. The patient enrolled in NCT06915454 (IRB# 242063) for intraoperative PET/CT imaging for real‐time surgical margin assessment. The patient also consented for 3D specimen scanning and virtual mapping (IRB #221597).

The patient then underwent left partial glossectomy. At the time of surgery, two separate 1.0 cm foci that were 1.0 cm apart were grossly evident on the mucosal surface of the left oral tongue (Figure 2A). For intraoperative specimen imaging, [18F]‐FDG (1.795 mCi) was administered 38 min before specimen extirpation, consistent with our institutional research protocol in which FDG is injected 30–90 min before specimen removal. The procedure was performed under an established institutional radiation safety workflow that included trained personnel, dosimetry and contamination precautions, radioactive waste segregation, and radiation surveys of the patient and specimen. A dedicated operating room and specialized surgical instruments were not required. Immediately following extirpation, the specimen was oriented and intraoperative FDG PET/CT scanning of the specimen was performed in the operating room using a high‐resolution mobile micro‐PET/CT device (XEOS Aura 10; XEOS Medical, Ghent, Belgium), with a reconstructed CT resolution of 0.1 mm and estimated PET transaxial spatial resolution of 1.87 mm. Imaging time was completed in under 15 min. All study procedures were performed with minimal to no disruption of standard operative workflow. PET/CT imaging demonstrated two hyperintense foci with maximum standardized uptake values (SUVmax) of 10.4 and 10.0, measuring 1.6 cm and 0.6 cm, respectively, separated by 7.9 mm of non‐avid tissue with an SUVmax of 6.4, and with uninvolved margins (Figure 2B).

**FIGURE 2:**
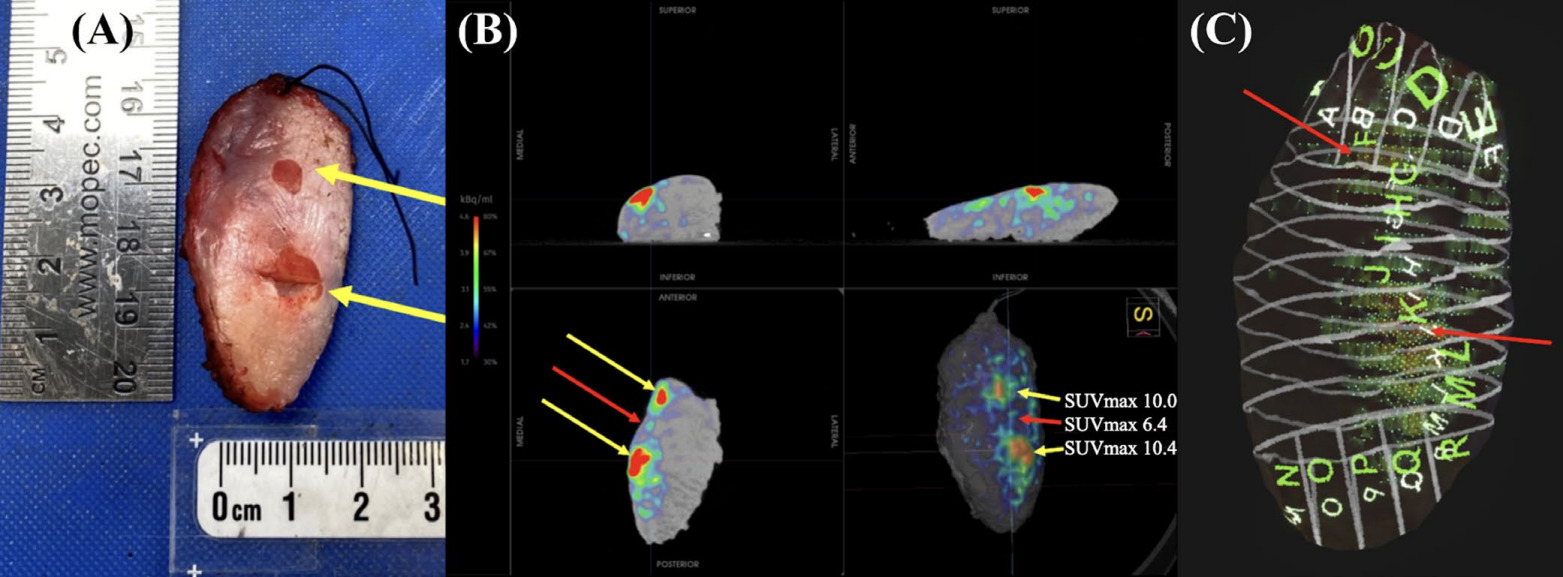
Post‐treatment surgical and imaging findings. (A) Gross photograph of the resected left oral tongue specimen (stitch anterior) demonstrating two distinct mucosal lesions (yellow arrows). (B) Intraoperative PET/CT scan of the left oral tongue demonstrating two hyperintense foci (yellow arrows) separated by a mildly avid intervening region corresponding to the inflammatory bridge (red arrow). (C) Fused intraoperative PET/CT and 3D specimen map demonstrating spatial co‐localization of metabolic activity with the annotated specimen model. Red arrows correspond to hyperintense foci.

The specimen underwent 3D surface scanning and virtual mapping per our team's previously published research protocol [6, 7, 8, 9]. Immediately following *en bloc* resection, the specimen's surface topography was captured using a structured light handheld 3D scanner (EinScan Pro HD, Shining 3D, Hangzhou, China) and reconstructed into a high‐fidelity virtual 3D model. During standard‐of‐care grossing, the inking scheme, submitted sections, and cassette identifiers were annotated on the 3D model using custom computer‐aided design annotation software, creating a digital twin of the pathologic processing (Figure 3A). The mapping confirmed that sections F, G, and H corresponded to the anterior lesion, sections I and J corresponded to the intervening bridge of inflammation without viable tumor, and sections K, L, and M corresponded to the posterior lesion, enabling precise spatial correlation between the regression pattern and its anatomic location within the specimen. The intraoperative PET/CT dataset was subsequently overlaid with the 3D specimen map to generate a fused model, enabling spatial correlation of metabolic activity with the annotated gross specimen and section‐specific histology (Figure 2C).

**FIGURE 3:**
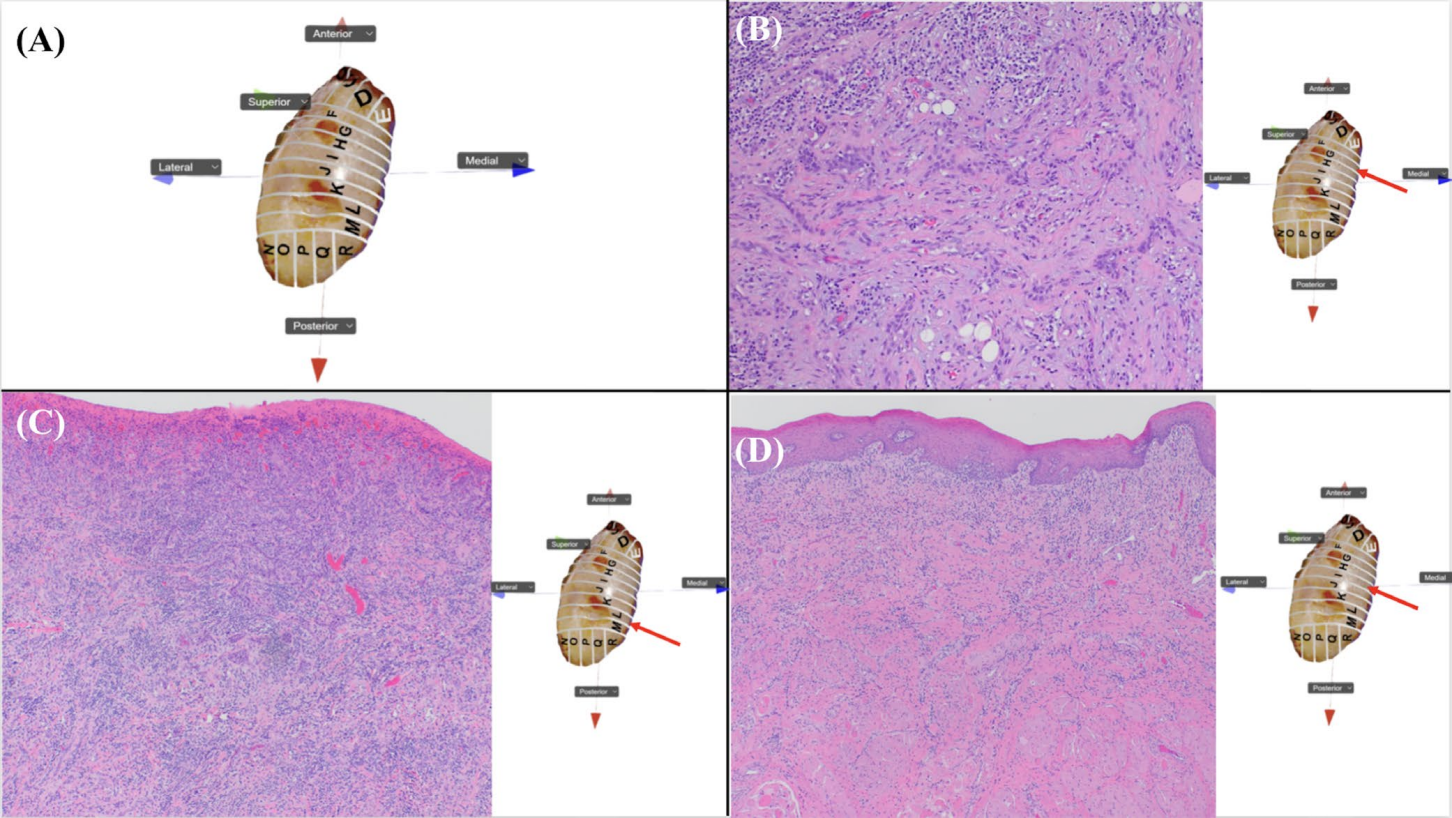
Histopathologic findings following neoadjuvant chemoimmunotherapy. 3D specimen map key with red arrow demonstrating corresponding section. (A) Three‐dimensional virtual model of the resection specimen with annotated grossing map. Each section submitted for histologic analysis is labeled corresponding to the cassette in the final pathology report. Sections F, G, and H correspond to the anterior lesion; sections I and J correspond to the intervening bridge of inflammation without viable tumor; sections K, L, and M correspond to the posterior lesion. (B) Hematoxylin and eosin (H&E) staining of the anterior lesion (representative section G) demonstrating invasive poorly differentiated keratinizing squamous cell carcinoma (pT2) with 0.2 cm depth of invasion and worst pattern of invasion (WPOI) of 1–4 (original magnification 10×). (C) H&E staining of the posterior lesion (representative section L) demonstrating poorly differentiated squamous cell carcinoma (pT1) with 0.9 cm depth of invasion and WPOI of 1–4 (original magnification 4×). (D) H&E staining of the intervening bridge (representative section I) demonstrating treatment effect with inflammation and no viable tumor (original magnification 4×).

Final pathology demonstrated two separate foci of residual poorly differentiated keratinizing SCC. Both lesions were on the left lateral tongue border. The anterior focus measured 0.5 cm with 0.2 cm depth of invasion (DOI) and worst pattern of invasion (WPOI) of 1–4 (Figure 3B). The posterior focus measured 1.8 cm with DOI of 0.9 cm and WPOI of 1–4 (Figure 3C). The two foci were separated by a 0.8 cm bridge of inflammation without viable tumor (Figure 3D), suggesting treatment‐induced tumor fragmentation. This pattern was consistent with a pathologic response, albeit with an atypical regression pattern. Final margins were negative, and there was no perineural or lymphovascular invasion.

## 3 Discussion

In this case report, we describe a patient with recurrent SCC of the oral tongue who underwent neoadjuvant chemoimmunotherapy prior to definitive surgical resection. Both intraoperative FDG PET/CT imaging and final histopathology revealed two separate foci of residual SCC separated by a bridge of inflammation without viable tumor, highlighting the heterogeneity of pathologic response to neoadjuvant chemoimmunotherapy. 3D specimen scanning and virtual mapping of the grossing process enabled precise spatial correlation of the two residual tumor foci and the intervening inflammatory bridge, facilitating the characterization of this regression pattern.

This report should be interpreted as a case study of an ongoing investigational application of intraoperative FDG PET/CT after neoadjuvant chemoimmunotherapy. Intraoperative PET/CT was used as an adjunct to standard intraoperative assessment, not as a substitute for direct surgical evaluation or frozen section analysis when indicated. Although preoperative PET/CT may have limited ability to characterize spatial regression patterns after neoadjuvant chemoimmunotherapy, intraoperative imaging as described in this manuscript was explored as a means of providing real‐time metabolic information during resection. This single case does not establish superiority over conventional methods or a definitive role in guiding resection extent.

As neoadjuvant chemoimmunotherapy is increasingly adopted in HNSCC, a recent systematic review and meta‐analysis of 23 prospective trials reported pooled major pathologic response plus complete response rates of 66% compared with 18% and 6% for dual‐agent and single‐agent immunotherapy, respectively [10]. With the majority of patients demonstrating some degree of pathologic response, the characterization of how residual disease is distributed spatially, not only how much remains, becomes increasingly relevant. However, preoperative FDG PET/CT has been shown to be unreliable in predicting pathologic response following neoadjuvant immunotherapy [5], leaving surgeons without a dependable tool to guide the extent of resection at the time of surgery.

Tumor regression patterns following neoadjuvant therapy have been characterized across other solid malignancies. In breast cancer, regression patterns following neoadjuvant chemotherapy have been classified as unifocal, limited multifocal, diffuse multifocal, or PCR, with diffuse multifocal regression associated with ipsilateral breast tumor recurrence after breast‐conserving surgery [11]. Interestingly, Zou et al. reported that non‐centripetal regression was more common following neoadjuvant chemoimmunotherapy (68%) than neoadjuvant chemotherapy alone (36%) in triple‐negative breast cancer [12]. If similar prognostic implications apply in HNSCC, the multifocal, non‐centripetal regression pattern observed in our patient may be associated with further recurrence risk and requires close postoperative surveillance. In esophageal cancer, fragmented regression patterns after neoadjuvant chemoradiation have been associated with higher pathologic staging, increased local recurrence, and poor overall survival [13, 14].

In HNSCC, the characterization of regression patterns in the neoadjuvant setting is beginning to emerge. A 2025 pilot study by Wang et al. used whole‐mount histopathology to classify regression patterns in 52 patients following neoadjuvant chemoimmunotherapy, identifying centripetal regression in 71.2% and non‐centripetal regression in 28.8% of cases [4]. Centripetal regression was subcategorized into complete (15.4%), unifocal (36.5%), and multifocal (19.2%) subtypes, while non‐centripetal regression included scattered (25.0%) and non‐regressive (3.8%) patterns [4]. PDL1 CPS ≥ 20 was significantly correlated with centripetal regression, and a > 50% post‐treatment PET/CT SUVmax reduction was predictive of centripetal response patterns [4]. Importantly, Wang et al. distinguished multifocal centripetal regression (foci < 5 mm from the core lesion) from scattered regression (foci ≥ 5 mm) based on the established 5 mm safe surgical margin for HNSCC, as scattered regression may make it unsafe to reduce surgical margins due to the risk of residual tumor foci beyond the margin boundary [4, 15, 16, 17]. Our patient's two residual foci of 1.8 and 0.5 cm with separation of 0.8 cm are consistent with a non‐centripetal scattered regression pattern as described in this classification system. However, our patient's PDL1 CPS of 90 with scattered regression differs with the pilot study's finding that CPS ≥ 20 predicted centripetal regression in 90.5% of cases. Beyond pattern classification, multifocal residual disease following neoadjuvant treatment may be associated with worse locoregional control compared to unifocal residual disease and has been shown to be an independent predictor of locoregional recurrence in HNSCC (HR 10.43; 95% CI 1.24–87.5) [18]. Our patient's scattered regression pattern reinforces the clinical significance of this regression pattern and need for close postoperative surveillance.

PD‐L1 expression as measured by CPS has become critical to treatment selection in HNSCC, particularly following its incorporation into NCCN guidelines for neoadjuvant pembrolizumab eligibility [3, 19, 20, 21, 22, 23]. However, CPS as currently applied describes the likelihood of a graded treatment response (i.e., complete, major, partial, none), but does not capture the spatial pattern of that response, which itself may hold prognostic value. Our patient had a CPS of 90 and did mount a pathologic response, yet demonstrated a scattered, multifocal pattern rather than expected centripetal regression per Wang et al. [4] This disconcordance underscores the need for intraoperative assessment tools that can characterize residual disease in real time, and our PET/CT protocol enabled accurate visualization of treatment response that correlated with final pathologic analysis. Future biomarker frameworks should consider incorporating spatial response characterization alongside graded response assessment.

This study has several limitations. As a single case report, the findings are descriptive and cannot establish the prevalence, reproducibility, or prognostic significance of the observed findings. However, the rarity of this presentation and its relevance to emerging literature on immunotherapy response patterns justify its reporting as a contribution to the growing evidence base. Additionally, this patient had recurrent disease following prior surgical resection without adjuvant radiation, which may alter the tumor microenvironment and local immune response, thus influencing the observed response pattern. Finally, the potential influence of concurrent chemotherapy on the observed regression pattern warrants consideration in this case. While platinum‐based agents and taxanes have been shown to enhance antitumor immunity, concurrent chemotherapy may also weaken the CD8+ T‐cell antitumor response activated by PD‐1 blockade compared to sequential administration [24, 25, 26, 27] In breast cancer, concurrent chemoimmunotherapy was associated with non‐centripetal regression compared to chemotherapy alone [12]. Our patient received paclitaxel and carboplatin alongside pembrolizumab, making it difficult to attribute the scattered regression to immunotherapy, chemotherapy, or their interaction. Future studies examining regression patterns should attempt to distinguish the contributions of each treatment modality where possible.

This case has implications for the management of locally advanced HNSCC in the perioperative immunotherapy era. First, the scattered regression pattern observed in our patient underscores that pathologic response assessment following neoadjuvant chemoimmunotherapy should extend beyond responder versus non‐responder classification. Kiong et al. concluded that tumor focality following neoadjuvant therapy in HNSCC should be routinely reported [18]. Our case, along with the regression pattern classification proposed by Wang et al. [4], supports extending this recommendation to include standardized spatial pattern characterization, similar to systems within breast [11], esophageal [13, 14], and rectal cancer [28]. Future studies should prospectively correlate these patterns with oncologic outcomes to determine whether regression pattern is an independent prognostic factor in HNSCC, as suggested by Kiong et al. [18] Second, the unpredictability of residual disease distribution for neoadjuvant therapy has direct implications for intraoperative decision‐making. Although response‐adapted surgery after neoadjuvant therapy is being explored, it remains investigational in HNSCC and careful discussion with patients preoperatively is required. In our patient, had surgical margins been aggressively narrowed based on the favorable radiographic response, residual disease may have been left behind. Given the demonstrated limitations of preoperative PET/CT in demonstrating pathologic response, intraoperative imaging may serve as a useful adjunct for surgeons in the neoadjuvant therapy setting [5]. In this case, intraoperative PET/CT correctly identified the two hyperintense foci and the intervening non‐avid inflammatory bridge, providing real‐time spatial information relevant to disease distribution and margin assessment that was not available through standard gross inspection or preoperative imaging alone, but corresponded exactly with final microscopic pathology. 3D specimen scanning with virtual grossing annotation and subsequent overlaying of PET/CT data, as performed in this case, offers a useful adjunct for mapping the pathologic findings within a resection specimen, and may support the development of regression pattern classification systems by providing reproducible spatial documentation of pathologic findings. Additionally, the established discordance between radiologic and pathologic regression patterns in breast cancer and emerging data in HNSCC further support the investigation of intraoperative imaging modalities for surgical decision‐making following neoadjuvant therapy [4, 12, 29, 30]. Given the high pooled pathologic response rates reported with neoadjuvant chemoimmunotherapy in HNSCC, intraoperative imaging tools and standardized reporting of regression patterns and tumor focality will become increasingly important as these regimens are adopted into routine practice [10].

## 4 Conclusions

This case illustrates that pathologic response to neoadjuvant chemoimmunotherapy in HNSCC can manifest as a scattered, multifocal regression pattern. These findings support the routine reporting of tumor focality and regression pattern following neoadjuvant therapy and highlight the potential role of intraoperative FDG PET/CT specimen imaging to characterize treatment response in this evolving clinical setting.

## Author Contributions

**Andreja Radevic:** conception and design, data analysis and interpretation, manuscript drafting, manuscript final approval. **Joaquin Austerlitz:** data analysis and interpretation, manuscript drafting, manuscript final approval. **Ashtyn McAdoo:** data analysis and interpretation, manuscript drafting, manuscript final approval. **Kyrionna Golliday:** data analysis and interpretation, manuscript drafting, manuscript final approval. **Takeshi Takahashi:** data analysis and interpretation, manuscript drafting, manuscript final approval. **Mitra Mehrad:** data analysis and interpretation, manuscript drafting, manuscript final approval. **Adam J. Rosenberg:** conception and design, manuscript drafting, manuscript final approval. **Barbara A. Murphy:** conception and design, manuscript drafting, manuscript final approval. **Eben Rosenthal:** manuscript drafting, manuscript final approval. **Michael C. Topf:** conception and design, data analysis and interpretation, manuscript drafting, manuscript final approval.

## Funding

This work was supported by the National Cancer Institute (5K08CA293255‐02).

## Disclosure

No material from other sources was reproduced in this study. All figures and data are original.

## Ethics Statement

This study was conducted with institutional review board approval (IRB #221597, IRB #242063). The study was conducted in accordance with the ethical standard of the Declaration of Helsinki. Written informed consent was obtained from the patient.

## Conflicts of Interest

The authors declare no conflicts of interest.

## Data Availability

The data that support the findings of this study are available from the corresponding author upon reasonable request.
